# Practical Notes and Formulæ

**Published:** 1886-05-20

**Authors:** 


### PRACTICAL NOTES AND FORMULÆ.

**Pruritus Vulvæ.**—In addition to appropriate systemic treatment, when indicated, local applications of hot water by sitz-bath or douche are recommended, followed by an application of the following:

℞. Amyli glyceriti........................30 grammes,
Zinc oxidi............................ 6 grammes,
Potassii bromidi...... ...................10 grammes,
Ext. cannabis indicæ...................... 2 grammes. M.

When acne accompanies the pruritus, the application night and morning of black soft soap for half an hour, followed by douche of water, and then by a lotion of strong infusion of black tea, has been tried with satisfactory results.—*Gazette de Gynecologie.*

**For Rheumatism.**—The following prescription is a good combination in rheumatism. I have tested it personally and know whereof I speak:

℞. Pot. iodide................................ʒ ijss,
Tr. cimicifugi...............................℥ ss,
Vin. colch. sem..............................℥ j,
F. E. henbane...............................℥ ss,
Simple syrup...............................℥ v.

M. Teaspoonful well diluted with water every four hours.—*N. E. Medical Monthly.*

**Elixir of Chloroform.**—

℞. Chloroform,

Tr. opii,

Tr. camphoræ,

Spts. ammoni aromat, } aa....................ʒ iss,

Ol. cinamon.................................m xx,

Brandy....... ...............................ʒ ij.

M. Dose from 10 to 40 drops.—*N. E. Medical Monhtly..*

**For the Typhoid Condition.**—No blunder is more common than to misconstrue into typhoid fever a typhoid *condition* of the system. In this state the bowels are extremely tender, tympanitic and inclined to frequent evacuations, despite the use of medicine. For this condition Dr. Batte , Tennessee, recommends the following formula:

℞. Sp. terebinth, resorcin, aa.......................ʒ vj,

Creasote...................................m xxiv,

Gum acaciæ...................................ʒ vi,

Aq. dest. ad.................................℥ vi.

S. A teaspoonful every four hours.—*Medical World.*

